# Pathway network inference from gene expression data

**DOI:** 10.1186/1752-0509-8-S2-S7

**Published:** 2014-03-13

**Authors:** Ignacio Ponzoni, María José Nueda, Sonia Tarazona, Stefan Götz, David Montaner, Julieta Sol Dussaut, Joaquín Dopazo, Ana Conesa

**Affiliations:** 1 Laboratory for Research and Development in Scientific Computing (LIDeCC), Department of Computer Science and Engineering, Universidad Nacional del Sur, Av. Alem 1253, Bahía Blanca, 8000, Argentina; 2 Planta Piloto de Ingeniería Química (PLAPIQUI), Universidad Nacional del Sur, CONICET, Bahía Blanca, CC717, Argentina; 3 Departamento de Estadística e Investigación Operativa, Universidad de Alicante, Alicante, 03080, Spain; 4 Computational Genomics Program, Centro de Investigación Príncipe Felipe (CIPF), Valencia, 46012, Spain; 5 Department of Statistics and Operations Research, University of Valencia, Valencia, 46010, Spain; 6 CIBER de Enfermedades Raras (CIBERER), Valencia, 46012, Spain; 7 Functional Genomics Node, (INB) at CIPF, Valencia, 46012, Spain

## Abstract

**Background**

The development of high-throughput omics technologies enabled genome-wide measurements of the activity of cellular elements and provides the analytical resources for the progress of the Systems Biology discipline. Analysis and interpretation of gene expression data has evolved from the gene to the pathway and interaction level, i.e. from the detection of differentially expressed genes, to the establishment of gene interaction networks and the identification of enriched functional categories. Still, the understanding of biological systems requires a further level of analysis that addresses the characterization of the interaction between functional modules.

**Results**

We present a novel computational methodology to study the functional interconnections among the molecular elements of a biological system. The PANA approach uses high-throughput genomics measurements and a functional annotation scheme to extract an activity profile from each functional block -or pathway- followed by machine-learning methods to infer the relationships between these functional profiles. The result is a global, interconnected network of pathways that represents the functional cross-talk within the molecular system. We have applied this approach to describe the functional transcriptional connections during the yeast cell cycle and to identify pathways that change their connectivity in a disease condition using an Alzheimer example.

**Conclusions**

PANA is a useful tool to deepen in our understanding of the functional interdependences that operate within complex biological systems. We show the approach is algorithmically consistent and the inferred network is well supported by the available functional data. The method allows the dissection of the molecular basis of the functional connections and we describe the different regulatory mechanisms that explain the network's topology obtained for the yeast cell cycle data.

## Introduction

The analysis of genome-wide transcriptomics data has changed in the last decade from a gene-centric vision, which evaluated thousands of gene expression changes in parallel, to a systems biology orientation where coordination among gene activities is pivotal. In light of this, data is analyzed from the perspective that genes do not act as independent entities, but as groups of cooperating molecules that define the cellular state [1,2]. Functional Enrichment [3] and Gene Set Enrichment Analysis (GSEA) [4], collectively denoted here as Enrichment Analysis (EA), are the paradigm of such vision. The EA relies on the definition of gene sets or pathways as blocks of genes that either share a cellular role or are sequentially connected to perform a given cellular function. EA methods have been developed with different adaptations to consider specific data structures such as regulatory networks [5], time series measurements [6], SNP data [7] or multifactorial designs [8], but they all attempt to identify gene sets whose global (de)activation is associated with the phenotype. Pathway databases such as KEGG, Reactome, BioCarta or the Gene Ontology host functional data and provide the annotation framework to define gene sets for enrichment analysis.

EA methods implicitly work under two assumptions. On the one hand, they consider that all genes in a gene set or a pathway equally contribute to the activity of that pathway; hence, the pathway is activated when a "sufficient" number of gene members is activated. This consideration does not take into account the differential regulatory factors that modulate each gene's participation in the pathway, such as different translation rates, enzymatic and complex-association kinetics or the quite versatile regulatory capacity of genes. An example of this last type is the heme biosynthesis pathway. This pathway involves eight enzymatic steps to transform succynil-coA and glycine into heme, the first being the synthesis of aminolevunilic acid by ALAS (aminolevunilic acid synthase), which is the committed step of the heme synthesis pathway and is usually rate-limiting for the overall pathway [9]. Hence, heme production is mostly controlled by ALAS regulation and not by a majority of pathway members. Furthermore, the variability in expression of human genes has been previously evaluated by our group across thousands of microarray experiments. The analysis demonstrated the constant expression of certain gene sets and we proposed a weighting scheme to account for the differential regulation capacity of genes within pathways [10]. Moreover, we have observed that gene regulation is associated with the network properties of the gene. Genes with a high cluster coefficient tend to show less pronounced variations at the transcript levels than those genes with lower connectivity [11] (Montaner, unpublished). All these examples illustrate the heterogeneous regulation capacity of genes within one pathway and their potentially differential contribution to its regulation.

The second assumption of EA methods is that pathways are generally considered as isolated boxes, and the interactions between them are normally not explored. However, pathways should be understood as a formalization of our understanding of cell biology and hence their boundaries are arbitrary or, actually, non-existing [12]. In fact, interconnections between genes and proteins go beyond pathway definitions and are condition dependent. Formal pathways may interact through either shared components (for example, purine and pyrimidine biosynthetic pathways share around 40 genes) or regulatory mechanisms (a pathway output might regulate or interact with proteins in a second pathway). Moreover, pathways may be connected by interaction elements that have not been discovered yet; for example, through regulation by non-coding RNAs such as miRNAs [13,14].

There are some recent examples in the literature of methodologies that analyze pathway interactions to understand the cross-talk between the functional blocks of a cellular system. Tools like ClueGO [15] and EnrichmentMap [16] display pathway connections by analyzing the overlapping between their annotated genes. Li and Agarwal [17] constructed a Pathway Consensus Network (PCN) from the physical interactions between proteins belonging to different pathways and used this global pathway interactome to map cancer genes in order to understand the progression of this disease. Huang and Li [18] extended this concept by incorporating gene expression data to define active protein-protein interactions and to identify phenotype-specific sub-pathway networks. More recently, Kelder *et al *[19] obtained additional links between pathways by searching for connecting paths that include not-yet-annotated proteins. Dutta *et al *[20] used the connectivity information in canonical pathway descriptions to identify study-relevant pathways and to characterize dependencies and connections among pathways using gene expression data. Liu *et al *[21] construct a pathway interaction network based protein-protein interactions and cellular pathways, which is applied to the identification of deregulated pathways as subnetworks using gene expression data.

In general, these methodologies rely on the selection of differentially expressed genes, enriched pathways and protein-protein interactions. Therefore, when two pathways have few described protein interaction links, but still functionally influence each other, their connection might be missed by these methods [17]. In this work, we present a novel approach to infer pathway interaction networks from gene expression data that relies on a new concept for understanding pathway activity and relationships. This approach considers the activation pathway as a coordinated and relevant change in the expression levels of some of their genes over a number of samples. Unlike EA methods, it does not explicitly require a majority of pathway genes being activated, but that some covariant expression profiles can be identified. The method defines a pathway level gene expression signature, or *profile*, that globally represents the main transcriptional regulation patterns within the pathway. Once pathway profiles have been defined, these are used to find connections between pathways. In this view, pathway links do not either depend on previous knowledge about protein-protein interactions or focus on identifying the genes shared between pathways, as it is the case with current pathway interaction approaches, but depend solely on pathway expression profiles.

In previous work, we used dimension reduction techniques to obtain pathway expression profiles, which are associated with a physiological outcome [22]. This reduction strategy has also been used in other scenarios to obtain pathway activity indexes linked to the toxicological properties of chemical compounds [23]. Pathway connections were obtained by a machine-learning method, which has been previously applied to identify gene networks [24]. We use well-studied data in the yeast cell cycle to demonstrate our methodology, discuss some relevant network links and provide guidelines to help interpreting the results. Finally we include an example of an Alzheimer gene expression dataset to illustrate how our method can be effective in revealing differential pathway connections associated to disease.

## Results

### The pathway network approach is numerically and biologically consistent

**Figure 1:**
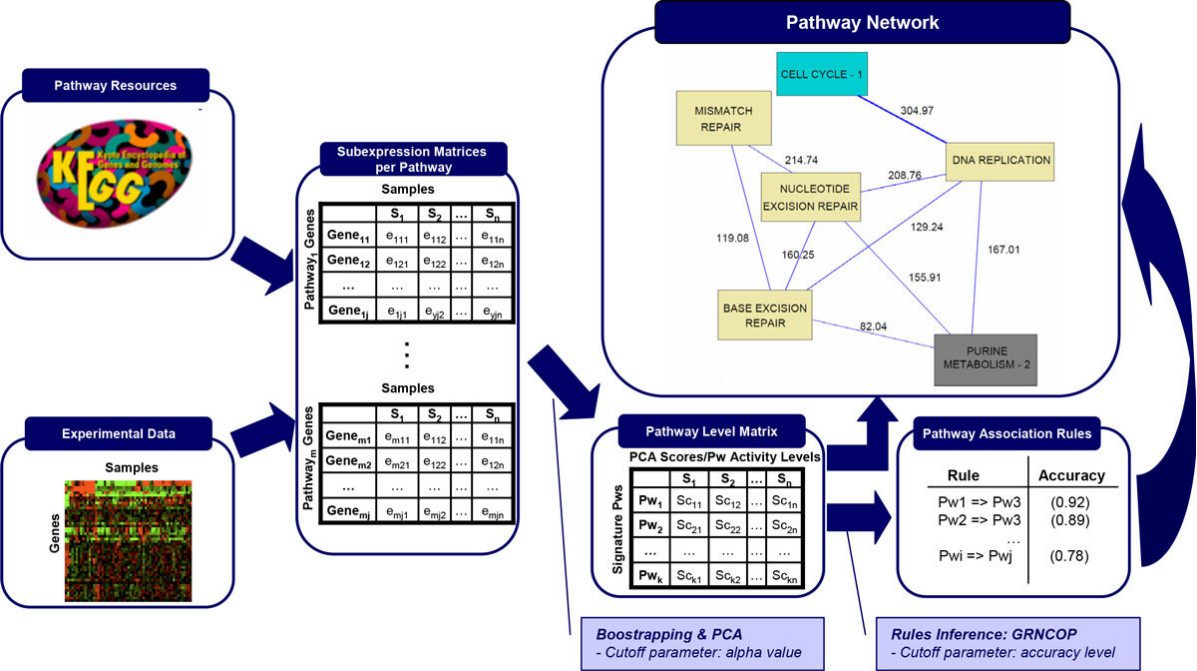
**Representation of the PANA algorithm**. Given a database of gene annotations into pathways and a gene expression experiment, an expression submatrix is generated per pathway by collecting the expression profiles of the genes annotated to each pathway. By applying PCA and bootstrapping to the expression submatrices, the Pathway Level Matrix is computed where row represent Pathway Profile. Next, a list of association rules between the pathway profiles is inferred by using GRNCOP. Finally, the pathway network is generated by selecting the rules with a high level of accuracy.

Our PAthway Network Analysis approach (PANA) consists of two basic steps (Figure 1). First, transcriptomics data is mapped to a pathway database to generate a set of gene expression submatrices, one per pathway, containing the expression values of the genes annotated to each pathway. Principal component analysis (PCA) is then applied to each submatrix to compress pathway information into a reduced number of expression profiles that characterize the pathways' gene expression changes. Numerically, these pathway profiles (PPs) are the scores of the principal components (PC) of the PCA, which are selected on the basis of a significance threshold *alpha*. The second step consists of obtaining a set of association rules that establish pair-wise connections between PPs. Direct and opposite rules are extracted, representing positive and negative correlation, respectively, between PPs. The quality value of the association rule is determined by its *accuracy*, which is basically a measure of the predictive performance of a rule in terms of the *sensitivity *and *specificity *metrics, commonly used in machine learning [25].

The performance of PANA approach was assessed both in terms of the formal characteristics of the inferred networks and in terms of functional consistency by comparing results obtained for the yeast cell cycle data against a database of yeast functional data.

### Evaluation of PANA network properties

#### Simulated datasets

**Figure 2:**
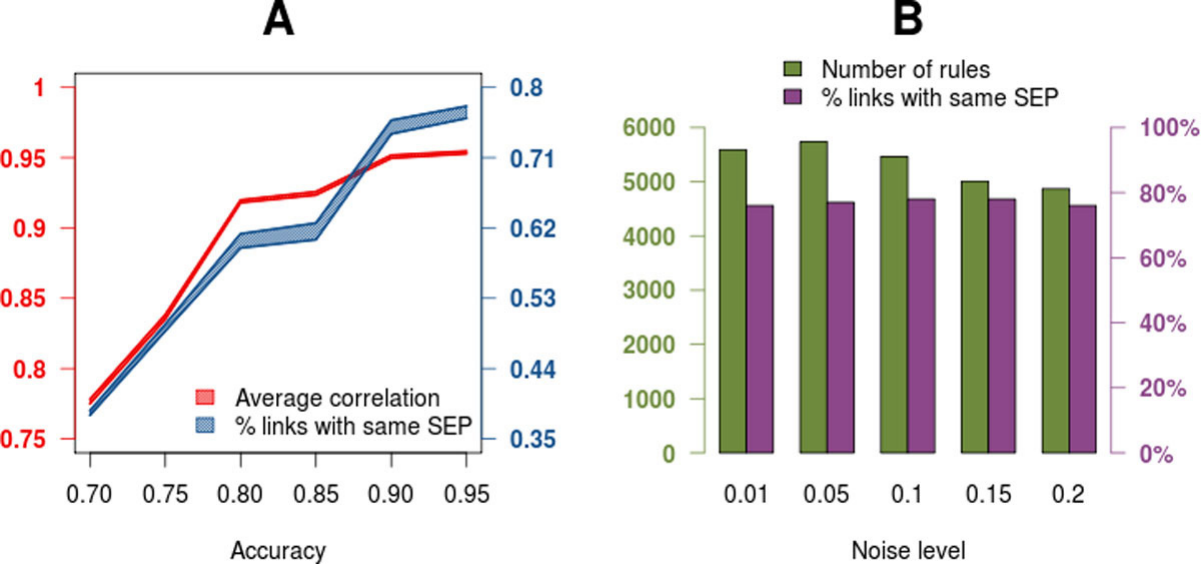
**Performance of PANA on synthetic data**. A) Performance of control parameters. Average correlation among network pathway profiles (left axis, red polygon) and percentage of links with same simulated expression profiles (right axis, blue polygon) as a function of the accuracy threshold. The upper and lower border of polygons indicate the range of variation at different alpha values. Noise level was set at 0.01. B) Robustness of PANA to experimental noise. Number of selected rules (left axis, red) and percentage of links with same simulated expression profiles (right axis, blue) as a function of the noise in the data. Alpha was set to 0.01 and accuracy to 0.9.

We used a simulated dataset to evaluate how different pathway factors, namely the number of genes in the pathway, the type of pathway profile and the percentage of pathway inner correlation (defined as the percentage of genes in the pathway that follow the main pathway profile) would affect the network results. The simulated dataset contained 24,990 genes and 36 samples. Pathways were defined as blocks of genes of different size. Each pathway was assigned a different simulated expression profile (SEP) out of seven possibilities (Additional file 1, Table S1) and also each pathway contained a different percentage of correlated genes. Table 1 shows the experimental factors and levels used to design the simulated datasets. In total, 245 different pathway designs were obtained by the combination of the three experimental factors and expression submatrices were generated for each of them using a multivariate normal distribution. The coefficient *s *used for the definition of the *sigma parameter *(the covariance matrix of this distribution) represents the level of noise in the generated data. The value of *s *was fixed in 0.01 for this experiment. We evaluated the performance of the PANA algorithm as a function its control parameters *alpha*, which modulates the extraction of PPs, and *accuracy*, that controls the identification of pathway links, and of the noise in the dataset defined by *s *(Figure 2).

**Table 1:** Experimental factors and levels used to design the simulated datasets.

Factors	Levels						
***Number of genes***	10	60	100	140	200		
***Type of profile***	1	2	3	4	5	6	7
***Inner correlation percentage***	0	0.2	0.4	0.5	0.6	0.7	0.8

First, PANA results were obtained on the synthetic data with a fixed *s *(0.01) value and varying levels of *alpha *and *accuracy*. Figure 2A shows the correlation value between PPs in extracted direct rules as a function of the PANA control parameters (left axis) and the percentage of rules that have pathways sharing the same SEP (right axis). As expected, increasing accuracy and decreasing alpha values associated with selected rules involving pathways with increasing correlation. For example, at an accuracy level of 0.9 and alpha of 0.05, the average correlation value in rules was 0.95 and 76% of the links involved pathways with the same SEP. In a second simulation experiment we evaluated the robustness of PANA to different noise levels. Figure 2B indicates that PANA finds relative constant number of direct rules at different noise levels (left axis), with slight decrease with ***s ***>= 0.1, and also a constant percentage of direct rules involving pathways with the same SEP (right axis). Similar observations were obtained when considering opposite rules (Additional file 1, Figure S1). From these results we concluded that control parameters on PANA correctly capture the correlation structure within the dataset and that the algorithm is robust to different levels of noise in the data.

#### The yeast cell cycle network obtained by PANA

The yeast cell cycle gene expression dataset used in the first experiment was published by Spellman *et al *[26]. This dataset contains microarray gene expression measurements at 24 time points of the yeast cell cycle synchronized by *cdc15*. We used this dataset to validate our methodology since it describes a well-known cellular system and there is extensive functional information available on yeast genes. The KEGG database was used as a pathway annotation scheme, and 112 pathways associated with the yeast genes were found.

Similarly to the results with the synthetic data, the number of pathway links inferred by PANA decreased with more restrictive alpha values and higher accuracy thresholds (Additional file 1, Table S2). Next, we reasoned that if the PANA methodology truly captures the functional links between pathways, the algorithm parameters will also control the biological consistency of the generated network. In order to evaluate the functional coherency of the different pathway network sets, the functional annotation data contained in the YeastNet2 database [27] were employed. This database contains 102,803 functional associations among 5,483 yeast genes. Each gene pair-wise relationship has an association score (AS), which integrates the degree of evidence obtained from the different types of data sources (gene co-citation in text mining, protein-based functional linkages, microarray expression correlations, and so on) in a normalized value. In our work, network validation was performed according to two AS metrics: the integrated AS, or the Bayesian AS (*b*AS) that uses a Bayesian method to integrate functional evidences; the AS obtained exclusively from microarray data, denoted here as the microarray AS (*m*AS). From these AS metrics, and given any pair of pathways *i *and *j*, the functional association strength among these pathways (*b*ASp*_ij _*or *m*ASp*_ij_*) was calculated in terms of the *b*AS and *m*AS of the pathway genes. A more detailed explanation about the YeastNet2 data and the method used for computing the *b*ASp and *m*ASp are provided in the Methods section.

**Figure 3:**
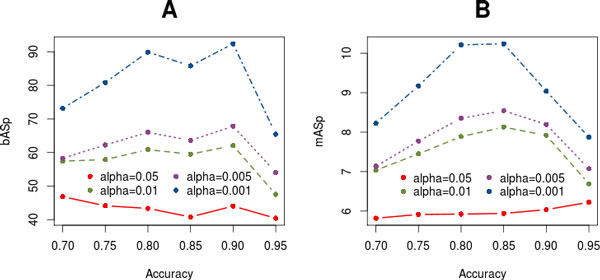
**Relationship between alpha and accuracy PANA parameters and the mean association strength of the resulting network, considering either the Bayesian *b*ASn (A) or Microarray *m*ASn (B) index, computed for the yeast cdc15 dataset**.

Figure 3 shows the relationship between the mean *b*ASp and *m*ASp values of the inferred networks, denoted as *b*ASn and *m*ASn respectively, and their alpha and accuracy values. The plot reveals that as the alpha value decreases, the *b*ASn of the identified pathway associations increases; i.e., the functional support of the inferred network is higher (Figure 3A). Regarding the accuracy parameter, the *b*ASn increases from 0.70 to 0.90, where the maximum value is reached. The fact that *b*ASn decreases in the highest accuracy range is a consequence of the reduced network size at these levels. When accuracy changes from 0.90 to 0.95, a few highly connected pathways drop, which has a major impact on the *b*ASn of the already sparse network. When the *m*ASn is considered, the relationship with the PANA parameters is similar, but the maximum value is reached at 0.85 (Figure 3B). The absolute values for *m*ASn are lower as this index exclusively uses evidence from the co-expression data.

Taken together, these results indicate that the two main control parameters of the PANA algorithm (the alpha value for the component selection in the first step and the accuracy value for rule inference in the second step) work consistently and are efficient in deriving pathway networks that are coherent with available biological information. From the obtained results, we selected the yeast cell cycle pathway network obtained with an alpha value of 0.001 and an accuracy value of 0.90 for further analysis. This network has been chosen because it has the highest *b*ASn value (92.37) and 252 associations. Therefore from this point onwards, any mention of the *yeast cell cycle PANA *refers to this particular network.

#### Functional significance of pathway links obtained by PANA

**Figure 4:**
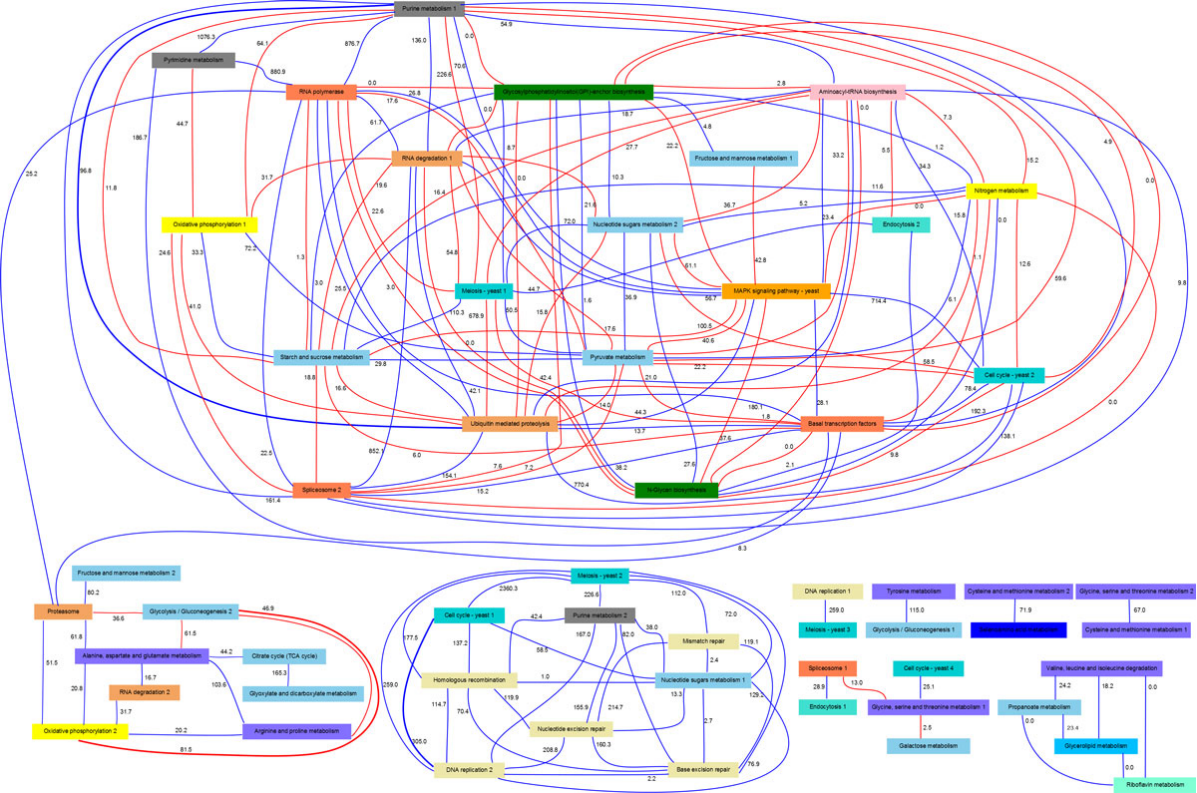
**The pathway network obtained from the yeast cdc15 dataset using an alpha value of 0.001 and an accuracy cutoff value equal to 0.90**. The pathway nodes filled with the same color belong to the same KEGG pathway category. The *b*ASp values are shown on the edges. Blue and red edges represent direct and opposite correlations, respectively. The thick links denote the pathway associations analyzed in detail. This network can be interactively browsed at http://pathwaynetworkanalysis.org.

The yeast cell cycle PANA (YCCPN) is presented in Figure 4. Additional information about YCCPN links, such as *b*ASp and *m*ASp values, the number of genes of the linked pathways, driving genes and other relevant information is included in the website of the PANA project at http://pathwaynetworkanalysis.org (see YCCPN website section). Several tests were designed to determine the relevance of the pathways associations inferred by the method. First, we asked whether the pathway links within the YCCPN provided greater functional evidence than expected by chance. For this purpose, the universal set of all possible yeast pathways associations and their *b*ASp values were computed from YeastNet2, resulting in 2,541 possible associations with a *b*ASp higher than zero. In Table 2, the distribution of the *b*ASp values in the universal set and the YCCPN are presented in terms of twelve different percentile values. From this table, it can be concluded that approximately 70% of the rules included in the YCCPN correspond to the first quartile of the universal set of pathway associations. Moreover, 40% of the YCCPN rules correspond to the 90% percentile of the universal set. Similar results were obtained when the network was compared to the *b*ASp values obtained from randomly generated gene blocks of the yeast genome (Additional file 1, Figure S2), indicating that the association strength of the rules in the YCCPN were significantly higher than what it would be obtained by random pairing of pathways.

**Table 2:** Comparison of the percentile breakdown of the bASp and mASp distributions for the universal set of pathways rules and YCCPN.

	10%	20%	25%	30%	40%	50%	60%	70%	75%	80%	90%	100%
***b*Univ.S**	1.28	2.03	2.40	2.89	4.41	6.51	9.70	**15.10**	19.09	23.76	**44.32**	2360.29
***b*YCCPN**	1.23	7.58	13.05	**16.64**	22.46	31.66	**44.30**	64.07	80.19	115.05	180.06	2360.29
***m*Univ.S**	1.43	1.56	1.66	1.78	2.08	3.28	4.07	6.14	7.07	**8.98**	16.70	130.93
***m*YCCPN**	0.00	0.00	0.00	0.00	0.00	1.53	3.89	**8.45**	11.23	15.72	28.15	130.93

Subsequently, the reference ASp value distribution analysis was repeated using the *m*AS values; i.e., the universal set of pathway associations integrated exclusively of the links supported by microarray data, resulting in 1,350 potential associations among pathways. When the *m*ASp distribution was compared to the YCCPN *m*ASp, we observed that approximately 30% of the links with the highest *m*ASp values included in the network corresponded to the 80% percentile of the universal set of pathway associations. Moreover, we have evaluated the number of rules with gene commonalities in the YCCPN and found that 76.19% of the associations corresponded to pathways that had no genes in common, indicating that the presence of shared genes between pathways is not the underlying mechanism of the inferred networks.

The conclusions of these analyses are two-fold. On the one hand, we demonstrate that the YCCPN contains a significant enrichment of pathway associations of strong functional links according to available biological knowledge. On the other hand, our approach, even when using only gene expression data, is able to capture relationships between pathways that are evidenced by other types of functional information and cannot be found by univariate co-expression analyses. This is supported by the fact that this enrichment is higher when *b*AS -collecting multiple sources of functional evidence- is considered instead of *m*AS (only microarray data). This claim is also consistent with the fact that the identified associations go beyond the presence of shared genes between pathways.

### Biological relevance of PANA results

We have demonstrated that the PANA approach unravels a network of connections between pathways, and that it is backed up by functional data. The next question is how these links can be interpreted in terms of their biological meaning. Our approach to this is the detailed analysis of the molecular function of the pathway *driving genes*. Driving genes are those genes that contribute the most to the definition of the pathway signature can be understood as fundamental pieces in their regulation (see Methods). We hypothesized that these genes can reveal the functional relationships between pathways and aid in the interpretation of the pathway network links. To help with this discussion, we refer readers to the PANA site where a fully hyperlinked YCCPN can be browsed. For notation purposes, driving gene names have been underlined in this section.

#### Cell cycle and DNA replication pathways

These two pathways are strongly associated (accuracy: 91.66%, *b*ASp: 304.97) and conform a cluster to other three DNA repair pathways. The connections between these processes are well documented by the literature and represent a suitable example to demonstrate the molecular fundaments of the pathway links. They share six genes corresponding to the MCM complex. However, none of those were selected as driving genes. Instead *CLB6*, *CDC45*, *MCD1 * (*ssc1*) and *RAD53 *included in Cell Cycle, and *POL30 * and *RFA1 * annotated to the DNA Replication pathway, were identified by minAS as major contributors to the connected pathway profiles. Note that *RAD53 * is annotated as DNA replication and DNA repair by other databases such as Saccharomyces Genome Database.

The regulation in eukaryotic cell cycle occurs during the transitions from the G1 to the S phase and from the G2 to the M phase [28]. These regulatory transitions strongly synchronize the cell cycle to DNA replication by means of several check-point proteins. Several of these proteins belong to the driving gene set of the cell cycle pathway, which explains the high *b*ASp obtained for this pathway link. For example, *CLB6 * stabilizes the S phase by promoting DNA replication while inhibiting other cell cycle activities. *CDC45 * is an essential protein for the initiation of DNA replication [29]. *MCD1 * is present during DNA replication and participates in the establishment of sister chromatid cohesion [30]. *MCD1 * is also required throughout the G2 and M phases to maintain cohesion [31]. Finally, *RAD53 * encodes a kinase, which is activated during DNA replication when DNA damage is detected. This kinase slows down the replication rate to promote DNA repair processes [32]. *POL30 * is the Proliferating Cell Nuclear Antigen (PCNA), a protein that acts as a processivity factor for DNA polymerase δ in eukaryotic cells. In response to DNA damage, this protein is ubiquitinated and involved in the *RAD6*-dependent DNA repair pathway.

**Figure 5:**
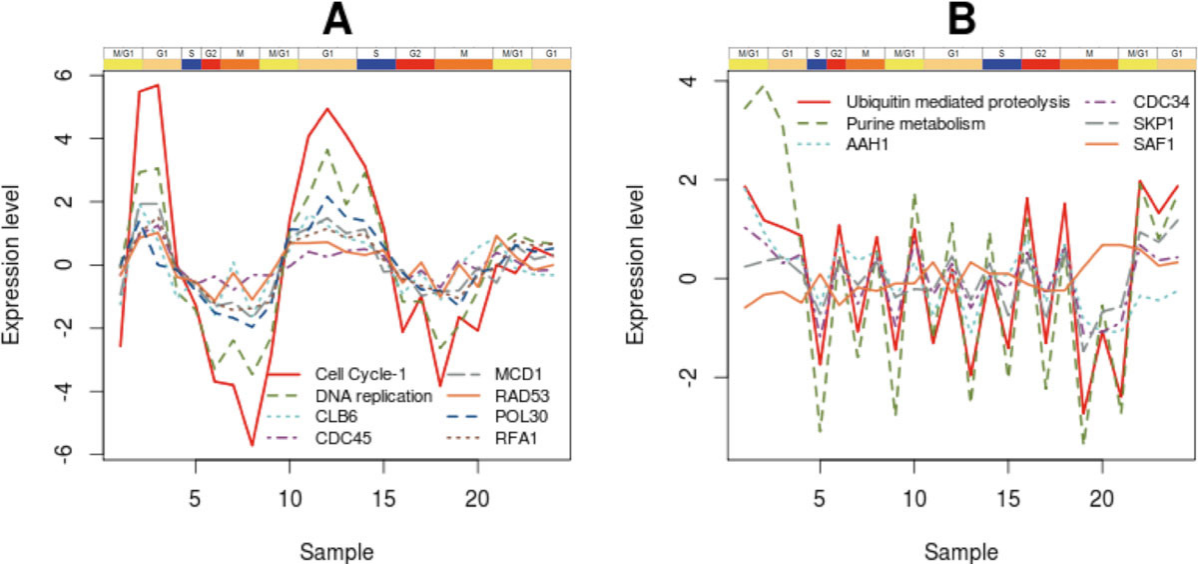
**Expression profiles of driving genes and pathways**. A) PPs for Cell Cycle and DNA replication pathways and expression profiles of *CLB6*, *CDC45*, *MCD1 *and *RAD53 *(Cell Cycle driving genes), *POL30 *and *RFA1 *(DNA Replication driving genes). All the genes are overexpressed in the G1 and the S phases simultaneously with DNA replication, and their activation patterns are consistent with their pathway temporal profiles. B) Gene expression for driving genes of the Ubiquitin-mediated Proteolysis (SAF1) and Purine Metabolism (*AAH1*, *CDC34*, *SKP1*) pathways. The *SAF1 *gene shows an inverse correlation to the expression of the *AAH1*, *CDC34 *and *SKP1 *genes.

Recent studies have identified strong correlations between genes *POL30 * and *MCD1 *[33]. In particular, they examined the expression of four genes (*MCD1*, *POL30*, *CLB2*, and *SUR7*), whose periodic expression during the yeast cell-division cycle is well established. From these experiments, the *POL30*-*MCD1 * pair achieved a higher level of correlation for synchronized (0.86) and unsynchronized (0.75) samples, proving that the simultaneous expression of both genes is an intrinsic feature of yeast growth. Therefore, there is clear evidence for strong temporal pattern matching between these driving genes. *RFA1 * is a subunit of Replication Protein A Complex (RPA). There is evidence for the regulatory action of *RAD53 * on RPA during the early S phase [34], and also on other proteins involved during DNA replication initiation. Therefore, all these genes play an important role in the synchronization between the cell cycle and the DNA replication process; hence their expression profiles are also closely matched. This is illustrated in Figure 5A, which depicts gene expression data for the driving genes, together with the pathway profiles of the Cell Cycle and DNA Replication pathways. Both pathway profiles are strongly correlated, like their driving genes, all of which show maximum activity in the G1 and S phases.

#### Glycolysis/gluconeogenesis and oxidative phosphorylation pathways

This association is an example of a negative relationship between two pathways; i.e., basically, their pathway profiles are negatively correlated. However, the link has a high *b*ASp value (81.47) and an accuracy of 100%. No genes are shared by the two pathways.

Eukaryotic cells produce energy in the form of ATP molecules by two different pathways: via glycolysis and by oxidation of glucose to ethanol or lactic acid. In particular, Cytochrome c-oxidase (COX), the terminal enzyme of the mitochondrial respiratory chain (MRC), plays a key role by regulating the rate-limiting step of respiration. This regulation mechanism facilitates aerobic ATP production. An analysis of the driving genes of the oxidative phosphorylation pathway signature, *COX12*, *COX13*, *COX17*, *CYT1*, *VMA4 *and *QCR6*, reveals the important roles of the genes associated with the cytochrome c-oxidase complex and ATP production in the conformation of the temporal profile of this pathway, indicating that COX activity and ATP synthesis are essential for the interpretation of this signature.

On the other hand, it is well known that the glycolytic flux is conditionally correlated with the ATP concentration in yeast. In particular, there is a strong negative correlation between glycolytic flux and intracellular ATP content; i.e., the higher the ATP content, the lower the glycolysis rate. Moreover, glycolytic enzymes *HXK2 *and *ENO1 *drastically reduce with an increasing flux [35]. When considering the glycolysis/gluconeogenesis signature pathway, the negative loading values of enzymes *HXK2 *and *ENO1 *and the identification of several glucose-repressed proteins as driving genes, such as *ACS1*, *GAL10 *and *ALD3*, suggest that this signature represents a situation where the glycolysis pathway is active, but reaching ATP saturation. This ATP concentration is promoted, in part, by the activity of the Oxidative Phosphorylation pathway, as indicated for the driving genes associated with COX and ATP syntheses. In other words, the negative link between Glycolysis and Oxidative Phosphorylation reflects the opposite involvement of ATP concentration in both pathways: while ATP production reflects the activity of the Oxidative Phospohorylation pathway through the action of its driving genes, high ATP levels down-regulate Glycolysis and modulate the expression of glucose-related genes. Note that this link is not explained by the presence of common genes between the pathways or protein-protein interactions, but by the action of a metabolic regulatory element.

#### Ubiquitin-mediated proteolysis and purine metabolism pathways

The previous two examples represent connections between pathways that are well established by the scientific literature. Here, we discuss a pathway association that might not be so evident. The link between ubiquitin-mediated proteolysis and the purine metabolism has a *b*ASp of 96.77 and an accuracy of 100%. Moreover in this case, no genes are shared by the two pathways.

Several driving genes were identified for these linked pathways. Specifically, we focus on the interaction and activation patterns of *SKP1 *and *CDC34 *from the Ubiquitin-mediated Proteolysis, and on *AAH1*, *POL32*, *RPA34 *and *RPC19 *from the purine metabolism pathway. *SKP1 *is an evolutionarily conserved kinetochore protein that forms part of multiple protein complexes, including the SCF ubiquitin ligase complex. *CDC34 *is an ubiquitin-conjugating enzyme (E2) and a catalytic subunit of the SCF ubiquitin-protein ligase complex (together with *SKP1*, *RBX1*, *CDC53*, and an F-box protein), which regulates cell cycle progression by targeting key substrates for degradation. *AAH1 *is the adenine deaminase-encoding gene and plays a central role in the salvage adenine pathway. There is a well-known relationship between the driving genes of the ubiquitin-mediated proteolysis and *AAH1*, which occurs during cell starvation. In response to nutrient limitation, *S.cerevisiae *cells enter a non-proliferating state termed quiescence. *AAH1 *is among the most tightly regulated genes upon entry into quiescence. Escusa *et al *[36] showed that *AAH1 *regulation at this stage is conducted by the gene *SAF1*, but its regulatory role is dependent on genes *SKP1 *and *CDC34*. Figure 5B presents the expression pattern of both these driving genes and *SAF1*, and reveals the negative correlation between *SAF1 *and *AAH1*, which is consistent with the negative regulatory role of the former on the latter. We postulate that this regulatory role of the ubiquitin-mediated proteolysis genes on key Purine Metabolism gene *AAH1 *might not be restricted to the quiescence process, but may operate during cell cycle progression, thus explaining the link between these two pathways.

The other driving genes of the Purine Metabolism are related with the synthesis, repair and degradation of DNA and RNA. In particular, *POL32 *codifies a third subunit of DNA polymerase δ, involved in chromosomal DNA replication and required for error-prone DNA synthesis in the presence of DNA damage. An association between *POL32 *and the ubiquitin system occurs during DNA replication and repair processes, where a small ubiquitin-related modifier (SUMO) and ubiquitin jointly affect a key signal integrator at the replication fork, PCNA [37,38]. Papouli *et al *[39] found that SUMO and ubiquitin cooperatively control the choice of pathway for the processing of DNA lesions during replication. This interaction is mediated by the recruitment of helicase *SRS2 *in order to inhibit DNA recombination; in particular, Pfander *et al *[40] presented evidence that *POL32 *SUMOylation is essential for the recruitment of this helicase. This result is concordant with other works [41,42], which suggested that the SUMO modification of yeast PCNA increases the activity of translesion DNA polymerase and inhibits a recombination-dependent bypass mechanism. Therefore, the overexpression of *POL32 *is consistent with a simultaneous activity of the ubiquitin-mediated proteolysis pathway.

### PANA to unravel differential pathway connections in disease

The yeast cell cycle analysis showed how PANA can describe pathway interconnections along a time course of events. In this section we evaluated how effective the PANA approach would be in studying differential pathway connectivity associated to a disease. For this, we used two microarray datasets generated for the study of Alzheimer Disease (AD). Both datasets were downloaded from the Gene Expression Omnibus (GEO) database http://www.ncbi.nlm.nih.gov/geo/ and were previously used by Dutta el al [20] for the detection of pathways associated with AD.

The first dataset (GEO ID: GDS810) [43], studied the expression profile of genes from the hippocampal region of the brain as a function of the progression of the disease (incipient, moderate, and severe). The second dataset [44] explored the effect of AD in six different brain regions: the entorhinal cortex, hippocampal field CA1, middle temporal gyrus, posterior cingulate cortex, superior frontal gyrus, and primary visual cortex (GEO ID: GSE5281). Since different regions of the brain are involved in controlling different biological processes, this dataset can provide insights into the tissue-specific activation of pathways. The entorhinal cortex region samples were obtained from patients in the early stages of AD, while the remaining samples were obtained from patients in the later stages of the disease. Dutta *et al *[20] specifically analyzed pathways that have statistically significant association with the AD pathway (KEGG hsa05010). The analysis focused on six conditions (moderate and severe samples in the disease progression dataset; and primary visual cortex, hippocampal field CA1, middle temporal gyrus, and posterior cingulate cortex regions in the brain regions dataset), where the AD pathway was found statistically enriched. Those pathways associated to the AD are at least 3 conditions were selected as relevant associations.

We have applied PANA to the same set of conditions. For the six cases, the pathway network corresponding to the disease and control samples were computed and contrasted, and we asked which pathways most frequently modify their association with the AD pathway when switching from healthy to disease status. The analysis revealed that PANA detects most frequent associations reported by Dutta, but in some cases with a lower frequency (Table 3). Notably, there were also new recurrent associations inferred only by our method. All new associations obtained by PANA that occur at least in three experiments are listed in Table 4. The last two columns contain the number of genes shared with the AD pathway and literature references that support the new associations [45-59].

**Table 3:** List of pathways more frequently associated with the AD pathway reported by Dutta *et al *[20].

Pathway	KEGG id	Shared Genes with AD pathway	Detected by PANA?
Gap junction	hsa04540	10	YES
GnRH signaling	hsa04912	20	YES
Huntington's disease	hsa05016	99	NO
Adherens junction	hsa04520	2	YES
Axon guidance	hsa04360	11	YES
Dorso-ventral	hsa04320	2	NO
Insulin signaling	hsa04910	10	YES
Long-term depression	hsa04730	11	YES
Long-term potentiation	hsa04720	29	YES
Neurotrophin signaling	hsa04722	12	YES
Oocyte meiosis	hsa04114	18	YES
Pathways in cancer	hsa05200	11	YES
Ubiquitin mediated proteolysis	hsa04120	0	NO

For comparison purpose, the third column shows the shared genes with the AD pathway and the fourth column indicates which associations are also detected by PANA.

**Table 4:** Pathways associated with the AD pathway obtained exclusively by PANA method.

Frequency	Pathway	KEGG id	Shared genes with AD pathway	Literature evidence
4	Citrate cycle (TCA cycle)	hsa00020	4	[46]
4	Pyruvate Metabolism	hsa00620	0	[47,48]
3	MAPK signaling	hsa04010	19	[49,50]
3	Peroxisome	hsa04146	0	[51,52]
3	VEGF signaling	hsa04370	11	[53,54]
3	Focal adhesion	hsa04510	5	[55]
3	Aldosterone-regulated sodium reabsorption	hsa04960	2	[56,57]
3	Carbohydrate digestion and absorption	hsa04973	2	[58,50,60]

Only those rules that occur at least in three experiments are listed.

A new rule is related with the Focal Adhesion pathway. Alzheimer's disease is a neurodegenerative disorder that results from a loss of synaptic transmission and ultimately cell death. The presenting pathology of AD includes neuritic plaques composed of beta-amyloid peptide (Aβ) and neurofibrillary tangles composed of hyperphosphorylated tau, with neuronal loss in specific brain regions. In the other hand, focal adhesion proteins assemble into intracellular complexes involved in integrin-mediated communication between the extracellular matrix and the actin cytoskeleton, regulating many cell physiological processes including the cell cycle. Remarkably, recent studies report that integrins bind to Aβ fibrils, mediating Aβ signal transmission from extracellular sites of Aβ deposits into the cell and ultimately to the nucleus. In particular, Caltagarone *et al *[54] discuss how the Aβ induced integrin/Focal Adhesion signaling pathways mediate in cell cycle activation and cell death during AD progression. Other novel association occurs with the Peroxisome pathway. In Alzheimer's disease lipid alterations are present early during disease progression. Some of these alterations point towards a peroxisomal dysfunction. Peroxisomes are present in all nucleated human cells, including all cell types of the brain, and perform anabolic and catabolic functions and play a major role in generation and decomposition of plasmalogens and docosahexaenoic acid. The levels of both of these lipids are decreased in brains of patients suffering from a generalized peroxisome biogenesis deficiency (Zellweger syndrome spectrum) and in AD. In particular, Kou *et al *[50] observed that the decrease in plasmalogens and the increase in VLCFA (very long-chain fatty acids) and peroxisomal volume density in neuronal somata showed a stronger association with neurofibrillary tangles than with neuritic plaques. Therefore, these results indicate substantial peroxisome-related alterations in AD, which may contribute to the progression of AD pathology. Another example is the link with the VEGF (vascular endothelial growth factor) pathway. VEGF, a critical mediator of angiogenesis, is present in the AD brain in the walls of intra-parenchymal vessels, in diffuse perivascular deposits, and in clusters of reactive astrocytes. In addition, intrathecal levels of VEGF in AD are related to clinical severity and intrathecal levels of amyloid-beta(Aβ). Emerging data support the idea that factors and processes characteristic of angiogenesis are found in the AD brain [52]. Rosenstein *et al *[53] also discuss about the role of VEGF in the perfusion deficits related with neurodegenerative disorders, such as Alzheimer and Huntington diseases, suggesting that problems in vascular tone regulation contributes to the pathogenesis of these disorders.

Interestingly, and as mentioned before, PANA was able to detect associations between pathways that only share few genes or even none (i.e. Peroxisome and AD pathways do not share genes). This contrasts with Dutta's results where the median number of shared genes linked to the AD pathway is 11. For PANA new rules this number drops to 3. This result is a direct consequence of fundamental differences between both algorithms. Dutta's method is oriented towards the topological information of the pathways (where the shared genes play a central role), whereas our methodology connects pathways based on their shared activity profiles. Still, the literature survey returns evidence of functional connections between AD and these pathways as discussed before.

## Discussion

Understanding the complexity of molecular interactions in a cellular system is one of the most challenging aspects of current genomics research. Many analysis approaches have been developed in recent years and have attempted to exploit functional information and multivariate analyses to provide answers about molecular systems functioning. These approaches rely on the systems biology concept; hence they analyze the collective behavior of groups of genes. In this work, we take one step forward by presenting a methodology that not only studies blocks of genes jointly, but also establishes relationships between these blocks. The result is a global, interconnected view of the system's transcriptional status.

There are some substantial differences between the PANA approach and most functional Enrichment Analysis methods. Probably, the most relevant one is the way that PANA extracts information from the gene set (or *functional block*). while the EA methods typically rely on identifying a significant majority of gene set members associated with the phenotype and consider all the genes equally contributing to the block's functionality, PANA is built upon the analysis of the correlation structure within the group of functionally related genes (for example, by forming part of a same sub-pathway, as exemplified in this work).

Both the covariation analysis and the feature extraction algorithm imply that the functional block hosts a level of transcriptional variation which is above a given noise threshold (see Methods) and that this might be concentrated in a subset of pathway genes. This procedure is able to address situations where pathways are roughly defined, include genes that are not necessarily co-expressed or when the regulation of the pathway is concentrated in a low number of switch genes. For example, the KEGG Purine Metabolism pathway (PMP) in yeast, present in our YCCPN results, includes reactions involving purine nucleotides and it branches out to histidine and thiamine metabolisms, sulfur assimilation and allantoin degradation pathways, among others. These other sub-pathways are not particularly seen as being highly regulated in our analysis. Additionally, *ADE4*, the first committed step in purine biosynthesis by catalyzing the reaction of PRPP, water and glutamine to 5'phosphoribosylamine, is identified as a driving gene in our analysis. *ADE4 * overexpression, but not the activity of other ADE genes, was found to increase purine biosynthesis in yeast [60]. PANA results are in agreement with these prominent regulatory roles of some pathway genes.

Another differential characteristic of the PANA approach is that the links between pathways do not derive from shared components or protein-protein interaction data, but exclusively from a co-transcriptional analysis. Co-expression has been largely used to infer gene regulatory networks [61-63], but these approaches normally ignore the participation of the genes in pathways and hence are limited in providing a global functional interpretation of the results [64]. This is the case of popular approaches such as WGCNA [65] -targeted to create scale-free networks from gene co-expression analysis- and GeneMANIA [66], focused in the integration of multiple gene association networks. We have shown that YCCPN connections are supported by functional evidence that goes beyond the gene expression data contained in the YeastNet2 database, and hypothesized that the pathway-centered multivariate analysis basis of our approach might be more robust in identifying functional transcriptional connections than pair-wise gene expression analyses. We have also shown in two examples that these transcriptional links can be explained by the action of molecular features that are not part of the connected pathways themselves. Such is the case of the Glycolysis/Gluconeogenesis and Oxidative Phosphorylation Pathways, which are regulated by ATP levels, and the Ubiquitin-mediated Proteolysis and Purine Metabolism Pathways, which are connected by the regulation of the *SAF1 *protein. This is an interesting property because it makes the approach amenable for application in situations of insufficient or misplaced pathway database annotation or when common regulatory elements are not proteins.

An unique PANA feature is the possibility of presenting different aspects of pathway behavior when the dimension reduction step results in multiple principal components being selected as pathway profiles. Each one can establish links with different pathways. For example, the Cell Cycle pathway is represented in the YCCPN by three profiles corresponding to principal component one, two and four of the PCA of the Cell Cycle gene expression matrix. Cell cycle_1 collects most of the canonical controllers of cell cycle progression and is linked to several DNA processing pathways, as discussed in the Results section. However, Cell cycle_4 presents a profile of activation at late time points of the yeast experiment. This profile is connected to the Glycine, Serine and Threonine Metabolism pathways, and indirectly to the Galactose Metabolism pathway (Figure 5). One of the driving genes of Cell Cycle_4 is *PHO85*, which negatively controls the expression of numerous genes induced under nutrient limitation conditions [67]. One of these repressed genes is *UGP1*, which catalyzes the reversible formation of UDP-Glc, a source compound in glycogen and trehalose biosynthesis. In our analysis, *UGP1 *is the driving gene of the Galactose Metabolism pathway and is negatively correlated with *PHO85*. Moreover, two driving genes in the Glycine, Serine and Threonine Metabolism pathways, *GCV2 *and *CHA1*, are related to nitrogen utilization under nutrient-limiting conditions. Hence the fourth profile of the Cell Cycle pathway might witness the coordination of this pathway with the nutritional state of the yeast cell.

Another interesting application of PANA is to unravel changes in pathway connectivity that associate to a given phenotype. This is relevant not only to understand the new functional status acquired in a disease situation, but also to explore possible side effects of treatments. Methods for differential molecular wiring have been described at the gene level [68,69] and have shown that differences in gene co-expression patterns rather than absolute expression level differences can determine phenotypic differences. In the Alzheimer dataset example we extend this concept to the pathway level and show that, by comparing the pathway network of healthy versus diseased individuals we can spot pathway connections that consistently change in Alzheimer patients. Some of these new connections can be detected by methods based on shared protein components but many other relevant ones were only found by our methodology.

PANA was developed in the microarray analysis context, but can be extended to other high-throughput methodologies provided that a functional database is available for feature annotation. The adaptative association rule algorithm, used for network construction, recommends evaluating the expression along a sufficient number of samples. This might preclude the utilization of this approach in reduced sample size experiments, but does not restrict the method to time series data. Besides, case control studies and multifactorial designs are potential experimental set-ups for PANA. On the other hand, the dimension reduction technique used in the first algorithm step, PCA, analyzes covariation across the entire data matrix. Other multivariate analysis approaches, such as biclustering or spectral analyses, might extend the possibilities of the method to identify the pathway profiles associated with a restricted number of samples and to fine-tune the network analysis to specific conditions within the experimental design.

In summary, we propose a novel method for the interpretational analysis of high-throughput data in systems biology research. This approach not only offers global views of the interconnections among the different functional blocks of the system, but also allows focusing on these links to reach the molecular basis of the network. We believe PANA is a useful tool to improve our understanding of the functional interdependencies that operate within complex biological systems.

## Methods

### The PANA algorithm

The proposed approach relies on the combination of dimensionality reduction methods with machine-learning techniques. Given a gene expression experiment and an annotation scheme for genes in pathways or functional modules, this method creates a gene expression submatrix for each pathway and uses a Principal Component Analysis (PCA) to reduce the dimensionality of the pathway expression data [22]. Each pathway will be represented by one or a few *pathway signatures *or *pathway profile *(PP), which collect most of the gene expression variation within the pathway and represent the pathway activity changes along the experiment. These PPs are used as input data to derive adaptive association rules based on mutual information maximization [24]. These rules can be seen as the covariation relationship between PPs and can be represented in the form of a network of pathway interactions with direct and opposite links depending on the direction of the rule. Hence, the network inference methodology consists of two main phases; pathway compression and association rule inference, which are described below.

#### Phase 1: Pathway compression

Given a transcriptomics experiment, let **X **be the expression data matrix of dimension *N *× *M*, where *N *is the number of genes measured and *M *is the total number of samples. Let x_nm _*n:1 ... N*, *m:1 ... M *be the expression value of gene *n *in sample *m*. Let F be the set of functional annotation (pathways) of the genes in the transcriptomic dataset. Let *N_f _*be the number of genes associated with each pathway *f ∈ *F.

1. For each f*∈ *F, create the expression submatrix of **X**, **X_f_**, with the *N_f _*rows corresponding to the genes associated with pathway ***f ***and with the same *M *columns as **X**. Obtain **X_f_^c ^**as the transposed, column-mean centered transformation of **X_f_**.

2. For each pathway *f*, obtain a number of pathway signatures *h_f_*, by applying a Principal Component Analysis- (PCA) based procedure that uses bootstrapping to obtain pathway signatures with a given confidence *alpha *according to the following procedure:

a) Given the original expression matrix **X **with *M *columns, sample *M *columns with replacement to obtain **X^r^**. Use **X^r ^**to calculate the variance of each gene. Approximate the gene variance distribution by a Gamma distribution as described [70] and obtain a gamma cutoff value as the 1-*alpha *percentile value of this distribution.

b) Apply PCA to each bootstrap pathway submatrix **X_f_^r ^**and select the principal components (PC) with variance (eigenvalues) higher than the gamma cutoff. Let PC_1_, PC_2_, ..., PC_k(r) _the selected PCs for matrix **X_f_^r^**, where 1 ≤ *k(r) *≤ rank(**X_f_^r^**).

c) Repeat 3 and 4 R times. Let H_f _be the set of all the selected PCs in the R repetitions: H_f _= {*1,..., k(1), 1,..., k(2), ..., 1,..., k(R)*}. Hence, each *i*∈H_f _has a frequency *q*_fi_. Select *i*∈H_f _with frequency *q*_fi _higher than a Q threshold (typically 95%) as the number *h_f _*of pathway signatures for pathway *f*.

3. Given the *h_f _*principal components selected using the criterion described above, the PCA decomposition of submatrix **X_f_^c ^**can be written as: **X_f_^c ^**= **T_f_P_f_^t ^**+ **E**, where **T_f _**is the scores matrix for pathway *f *(with dimensions *M *× *h_f_*), **P_f _**is the loadings matrix for pathway *f *(with dimensions *N_f _*× *h_f_*) and **E **is an error term. The scores matrix **T_f _**represents the *h_f _*pathway signatures for pathway *f*. These new *h_f _*functional variables represent the coordinative expression patterns of the genes associated with pathway *f*. **P_f _**collects the contribution of each gene to each pathway signature.

4. Create a pathway level matrix (**PLM**) through the row-wise concatenation of the **T_f _**scores matrices of all the pathways with at least one selected pathway signature. Hence, all the pathway signatures selected during Step 5 are included in the **PLM**, which has ∑_*f*∈F _*h_f _*rows and *M *columns.

**Figure 6:**
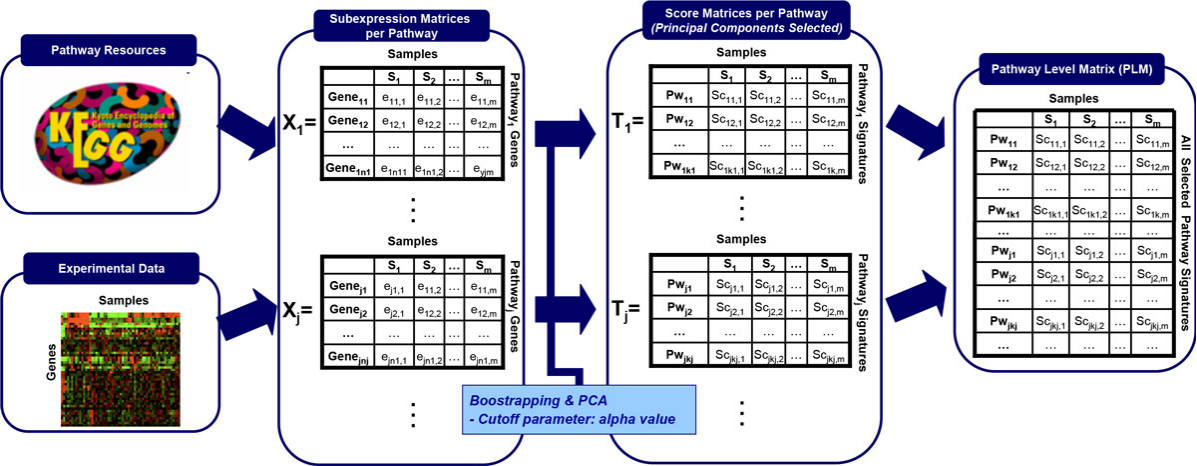
**Detailed representation of Phase I of the PANA algorithm: generation of the Pathway Level Matrix (PLM)**.

Phase 1 is depicted in Figure 6.

#### Phase 2: Inference of association rules

1. For each pathway signature, make an adaptive discretization of **PLM **into two states: *high *and *low activity levels *of the pathway signature, represented by values 1 and -1, respectively. For example, a relative high PCA score for a sample in a pathway signature means that the group of genes associated with this pathway are, in general, over-expressed in that sample. An adaptive method based on the partition entropy metric, typically used in Information Theory, is employed for data discretization [24]. The discretization procedure works as follows: discretization of the **PLM **is computed per pathway signature *j *(row *j *of matrix **PLM**). The discretized matrix obtained for the **PLM **and pathway signature *j *(**PLM_j_**) is denoted as **δPLM_j_**. For the computation of **δPLM_j _**a set of discretization thresholds (***t*_lj_**) for each signature pathway *l *(**PLM_l_**), with *l* ≠ *j*, is calculated in relation to **PLM_j_**. The algorithm for computing ***t*_lj _**considers each score value shown by the pathway signature **PLM_l _**as a candidate threshold ***t*_c_**. Therefore, for each possible ***t_c _***value, the sample set **PLM_l _**is partitioned into two subsets, namely *S*_-1 _and *S*_1_. *S*_-1 _contains all the samples where **PLM_l _**has a score value that is less than or equal to ***t*_c_**, whereas *S*_1 _contains all the samples where **PLM_l _**has a score value that is greater than ***t*_c_**. In other words, *S*_-1 _and *S*_1 _represent sample sets (columns of the **PLM**) where **PLM_l _**has low and high activity levels, respectively, on the basis of ***t*_c_**. Next, calculate the partition entropy, which is a statistical indicator of the quality of threshold ***t*_c _**as a discretization value for **PLM_l _**in relation to the discretization of **PLM_j_**. The partition entropy is computed from the discretized values of rows **PLM_l _**and **PLM_j_**, where **PLM_l _**is discretized using ***t*_c_**, while **PLM_j _**is discretized using its average score value. In numerical terms, the value returned by this metric is a real number between 0 and 1. When the partition entropy value associated with a discretization approximates 0, the threshold ***t*_c _**that generates this discretization represents a better solution. Consequently, the ***t*_c _**that minimizes the partition entropy is selected as ***t*_lj. _**Details about the equations for the computation of the entropy and partition entropy metrics can be found in Mitchel [71] and Kohani [72], respectively.

2. Extract the association rules from the discretized matrix by detecting covariation between pairs of pathway signatures. The rules inference procedure is applied to each **δPLM_j _**in order to determine which pathway signatures are linked with pathway signature *j *(**PLM_j_**). This task is carried out by a classifier optimization method [24], which infers association rules and their accuracy values. In mathematical terms, the classifiers are computed as solvers of the following combinatorial optimization problem:

$$\overset{k}{\underset{j=1}{⋃}}\underset{{\overset{¯}{\pi }}_{j}\in \text{P}}{\max}\;\sigma \left({\overset{¯}{\pi }}_{j},\;\delta \;PLM_{j}\right)$$

Where:

• *k *= number of pathway signatures in **PLM **(*k* = ∑_*f*∈F _*h_f_*)

• **P **is the space of all the vectors ***v ***of dimension *k*, so that *v_i _*represents a class of association rule ∀*i*, *i *= 1 ... *k*,

• **δPLM_j _**is the discretization of the PLM for pathway signature *j*,

• ${\overset{¯}{\pi }}_{j}\in P$ is a classifier of all the rules with an incidence on pathway signature *j*,

• (${\overset{¯}{\pi }}_{j}$), **δPLM_j _**is a performance function that evaluates the accuracy of ${\overset{¯}{\pi }}_{j}$ a classifier obtained from the **δPLM_j _**data,

Therefore, per each pathway j, the inference method obtains the pathways linked with j by solving this optimization problem by combinatorial analysis. Rule accuracy is computed for the **σ **function in terms of the well-known sensitivity and specificity metrics by using the equation proposed by Carballo and Freitas [25]. Therefore, those rules with accuracy values above a predefined threshold are selected for the network construction. The resulting network represents the relationships between pathway signatures and has directionality. The direction of a network link represents the direction in which the association rule holds and indicates logical causality.

**Figure 7:**
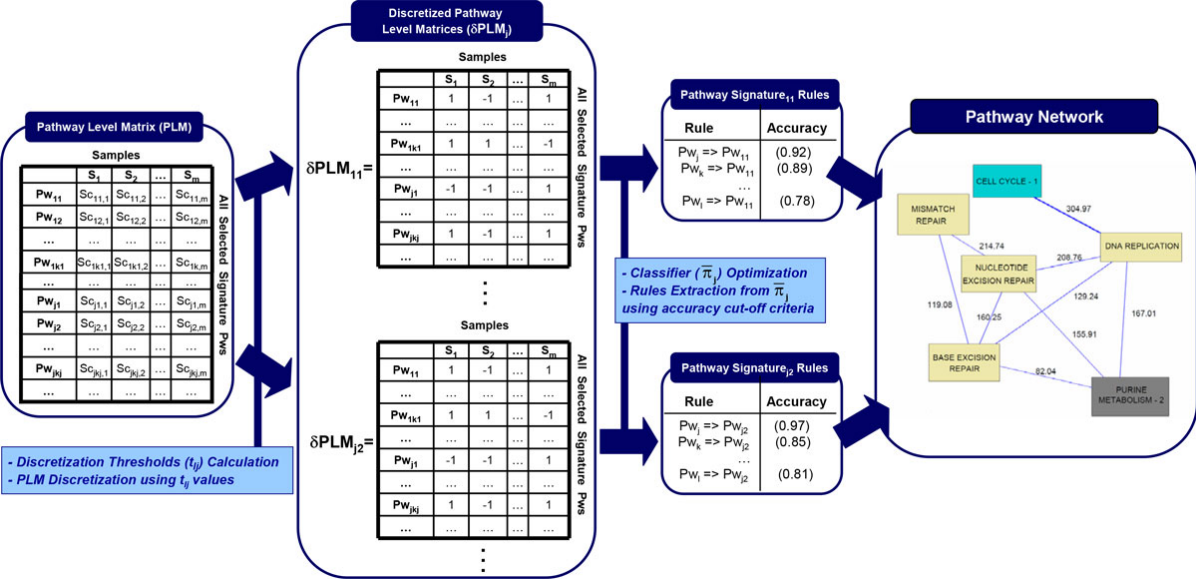
**Detailed representation of Phase II of the PANA algorithm: Inference of Association Rules from the PLM**.

Phase 2 is schematized in Figure 7.

### Determination of *driving genes*

In order to better interpret the associations between pathways, we propose to determine which genes contribute most to create the *pathway profile *(PP). As PPs are modeled by PCA, loadings represent the contribution of each gene to the definition of the PP. Genes with low loadings are poorly correlated with the pathway profile, while those with high loadings are highly correlated. Frequently, a subset of pathway genes can be identified as being mainly responsible for the definition of the PC. We will refer to these genes as *driving genes *as they are "pulling" the pathway signatures for having the greatest weights in the PC.

To identify driving genes, PANA uses the minAS method [70], which is an algorithmic strategy to classify features according to the values of a statistic that measures the importance of those features in the model. In our case, the model is a PCA and the statistics are the gene loadings. Usually, in PCA models, each PC is defined by a relatively low number of variables while most of the variables will have loadings close to zero. Accordingly, minAS works under the assumption that the distribution of the statistic is at least bimodal: it follows a mixed distribution with at least two components. The first component (typically with the highest mode) is associated with variables with a negligible value of the statistic which are normally of no interest, while the rest of components correspond to variables where the statistic has high values. minAS obtains a cutoff value that separates the first component from the rest by firstly estimating the density function of the statistic with a Kernel Density Estimator and by then computing the point where the first local minimum is reached. Hence this cutoff is not arbitrary, but consistent with the information contained in the data [70]. Those genes with absolute loading values higher than the minAS cutoff will be selected as the *driving genes *of each pathway profile.

### Yeast Cell Cycle dataset

The microarray data used for the inference of *yeast cell cycle PANA *(YCCPN) were published by Spellman *et al *[26]. These expression values were obtained for *S. cerevisiae *cell cultures, which were synchronized by three different methods: the cdc15, cdc28 and alpha factors. The data-transformation method used by Spellman *et al *returned background-corrected signal log ratios, with control as an average expression level extracted from asynchronous cultures of the same cells growing exponentially at the same temperature in the same medium. For this work, the cdc15 experiment was selected as the benchmarking dataset for the generation of YCCPN because it contains the largest number of data points (24 samples), thus providing the largest number of instances for the machine-learning method. In this time series, cell cycle progression was blocked at a specific point by conditional factor CDC15 which, if removed, permits cells to recommence progression through the cell cycle in a synchronous fashion.

### Yeast Cell Cycle PANA Network validation

To validate YCCPN, the functional annotation data contained in YeastNet2 [27] were used. Approximately 1800000 individual experimental observations were integrated into YeastNet2 from ten different types of functional genomics, proteomics and comparative genomics datasets by optimizing a total of ~155 free parameters to construct the whole network.

YeastNet2 contains a total of 102803 links covering 5483 yeast proteins, this which represents 95% of the validated yeast proteome and provides an association score (AS) for each pair-wise gene relationship. AS values are obtained from each kind of experimental evidence separately (i.e, gene co-citation in text mining, protein-based functional linkages, microarray expression correlations, etc) and jointly combining, following a Bayesian method, different evidence AS into an unique AS value per gene association.

In our work, network validation was performed according these two AS metrics: the integrated AS value, named Bayesian AS (*b*AS) represents the amount of scientific evidence of a putative pathway association by taking into account all the biological data sources, and the AS value obtained from microarray evidence exclusively, named Microarray AS (*m*AS). From both AS metrics, and given a pair of pathways i and j, the functional association strength between these two pathways (*b*ASp_ij _or *m*ASp_ij_) was computed as the sum of the functional association scores of all the gene pairs which can be established between these pathways. Moreover, a mean *b*AS and *m*AS value for each network were calculated as the average *b*ASp and *m*ASp values, respectively, of its pathway associations. Although ASp is a summing-up value, there was no correlation between the magnitude of ASp and the size of the pathway involved (Additional file 1, Figure S3).

### The PANA project website

The YCCPN described in this paper can be visualized and explored on the project web site at http://pathwaynetworkanalysis.org. On this web site, some fundamental definitions - such as a pathway signature and driving gene concepts - are enunciated, and the pathway network is depicted in a web-navigable format. The YCCPN figure includes zooming capabilities to improve dynamic visualization. Each network node -pathway signature- is linked to a gene expression painted image of the pathway obtained with the Paintomics tool [73], which has further links to the KEGG data. These images also highlight the driving genes by bold-lined boxes. These boxes usually have more than one associated gene. For this reason, a pop-up window is displayed for each box, where the driving genes are denoted by blue filled squares. The network edges discussed in this paper are indicated by thick lines with hyperlinks to a text document explaining the biological background of the association.

The pathway signatures of YCCPN are also available as a pdf file in the section *Additional Information*. In this document, three plots are included for each pathway signature: the pathway signature profile, the loading value curve (with an identification of the gene with the highest loading value), and the temporal profile that corresponds to this gene. This information is useful for understanding the direction of the principal component which represents the signature pathway. Finally, the annotation of samples at cell cycle phases can be found in the section *Additional Information*. In particular, the cdc15 dataset contains 24 samples obtained during 300 minutes [74].

## Competing interests

The authors declare that they have no competing interests.

## Authors' contributions

IP and AC designed the computational methodology, conceived the study and drafted the paper. MJN designed the method for generating simulated data. ST obtained driving genes by adapting the minAS algorithm. SG contributed to the graphical representation of the YCCPN. DM performed the variability analysis. JSD created the project web site. JD helped in drafting the manuscript. All the authors read and approved the manuscript.

## Supplementary Material

Additional file 1 **Figure S1. Performance of control parameters for opposite rules**. Average correlation among Pathway Profiles of opposite rules (left axis, red polygon) and percentage of opposite links with same simulated expression profile SEP (right axis, blue polygon) in the resulting network, as a function of the accuracy threshold. The upper and lower border of polygons indicate the range of variation at different alpha values. Noise level was set at 0.01. **Figure S2. Association score enrichment against random pathways**. For each pathway association rule R present in the YCCPN, a total of 100 random pathway associations with the same cardinality pattern as R were generated, the ASp values computed and the percentile position of the ASp of R in its reference distribution was obtained. The cardinality pattern of a rule is defined by three values: the amount of genes contained in each pathway linked by the rule, and the number of shared genes between both pathways. This analysis revealed that most (63% of the links) of the rules obtained by the PANA method are located in the 20% percentile of the 100 random trials of their gene cardinality pattern. In particular, the average *b*ASn of network integrated by the random links is low (28.50) in comparison with the *b*ASn of the YCCPN (123.61). The difference between YCCPN *b*ASn and the random *b*ASn was statistically significant (t-test p-value < 0.05). **Figure S3. Independence of the ASp score of the pathway size**. Relationship between pathway association scores (*b*ASp and *m*ASp values) and the number of genes in the left (dot) and right (cross) pathways. Lack of correlation is observed in all cases. **Table S1. Simulated expression profiles (SEP)**. Temporal expression patterns defined for the generation of simulated time series for the artificial pathways. **Table S2**. **Network size (number of pathway associations) inferred using different accuracy and alpha values in the Yeast Cell Cycle network obtained by PANA**. Click here for file

## Declarations

The publication costs for this article were funded by the FP7 STATegra project, grant 306000.

This article has been published as part of *BMC Systems Biology *Volume 8 Supplement 2, 2014: Selected articles from the High-Throughput Omics and Data Integration Workshop. The full contents of the supplement are available online at http://www.biomedcentral.com/bmcsystbiol/supplements/8/S2.

## References

[B1] Barabasi AL Oltvai ZN Network biology: understanding the cell's functional organization Nat Rev Genet 2004 5 101 113 10.1038/nrg1272 14735121

[B2] Dopazo J Functional interpretation of microarray experiments OMICS 2006 10 398 410 10.1089/omi.2006.10.398 17069516

[B3] Al-Shahrour F Minguez P Tárraga J Medina I Alloza E Montaner D Dopazo J FatiGO +: a functional profiling tool for genomic data. Integration of functional annotation, regulatory motifs and interaction data with microarray experiments Nucleic Acids Res 2007 35 W91 6 10.1093/nar/gkm260 17478504 PMC1933151

[B4] Mootha VK Lindgren CM Eriksson KF Subramanian A Sihag S Lehar J Puigserver P Carlsson E Ridderstrale M Laurila E PGC-1alpha-responsive genes involved in oxidative phosphorylation are coordinately downregulated in human diabetes Nat Genet 2003 34 267 273 10.1038/ng1180 12808457

[B5] Shojaie A Michailidis G Analysis of gene sets based on the underlying regulatory network J Comput Biol 2009 16 407 26 10.1089/cmb.2008.0081 19254181 PMC3131840

[B6] Nueda MJ Sebastián P Tarazona S García-García F Dopazo J Ferrer A Conesa A Functional assessment of time course microarray data BMC Bioinformatics 2009 10 Suppl 6 S9 10.1186/1471-2105-10-S6-S9 19534758 PMC2697656

[B7] Fridley BL Biernacka JM Gene set analysis of SNP data: benefits, challenges, and future directions Eur J Hum Genet 2011 19 837 43 10.1038/ejhg.2011.57 21487444 PMC3172936

[B8] Montaner D Dopazo J Multidimensional gene set analysis of genomic data PLoS One 2010 5 4 e10348 10.1371/journal.pone.0010348 20436964 PMC2860497

[B9] Ponka P Cellular iron metabolism Kidney Int 1999 55 Suppl 69 S2 11 10.1046/j.1523-1755.1999.055suppl.69002.x 10084280

[B10] Montaner D Minguez P Al-Shahrour F Dopazo J Gene set internal coherence in the context of functional profiling BMC Genomics 2009 10 197 10.1186/1471-2164-10-197 19397819 PMC2680416

[B11] Minguez P Dopazo J Assessing the biological significance of gene expression signatures and co-expression modules by studying their network properties PLoS ONE 2011 6 e17474 10.1371/journal.pone.0017474 21408226 PMC3049771

[B12] Schaefer CF Anthony K Krupa S Buchoff J Day M Hannay T Buetow KH PID: the Pathway Interaction Database Nucleic Acids Research 2009 37 D674 D679 10.1093/nar/gkn653 18832364 PMC2686461

[B13] McCarthy N Epigenetics: Layer by layer Nat Rev Cancer 2011 11 830 22048565 10.1038/nrc3172

[B14] van Kouwenhove M Kedde M Agami R MicroRNA regulation by RNA-binding proteins and its implications for cancer Nat Rev Cancer 2011 11 644 56 10.1038/nrc3107 21822212

[B15] Bindea G Mlecnik B Hackl H Charoentong P Tosolini M Kirilovsky A Fridman WH Pagès F Trajanoski Z Galon J ClueGO: a Cytoscape plug-in to decipher functionally grouped gene ontology and pathway annotation networks Bioinformatics 2009 25 1091 1093 10.1093/bioinformatics/btp101 19237447 PMC2666812

[B16] Merico D Isserlin R Stueker O Emili A Bader GD Enrichment Map: A Network-Based Method for Gene-Set Enrichment Visualization and Interpretation PLoS ONE 2010 5 e13984 10.1371/journal.pone.0013984 21085593 PMC2981572

[B17] Li Y Agarwal P A Pathway-Based View of Human Diseases and Disease Relationships PLoS ONE 2009 4 e4346 10.1371/journal.pone.0004346 19194489 PMC2631151

[B18] Huang Y Li S Detection of characteristic sub pathway network for angiogenesis based on the comprehensive pathway network BMC Bioinformatics 2010 11 S32 10.1186/1471-2105-11-S1-S32 20122205 PMC3009504

[B19] Kelder T Eijssen L Kleemann R van Erk M Kooistra T Evelo C Exploring pathway interactions in insulin resistant mouse liver BMC Syst Biol 2011 5 127 10.1186/1752-0509-5-127 21843341 PMC3169508

[B20] Dutta B Wallqvist A Reifman J PathNet: a tool for pathway analysis using topological information Source Code for Biology and Medicine 2012 7 10 10.1186/1751-0473-7-10 23006764 PMC3563509

[B21] Liu KQ Liu ZP Hao JK Chen L Zhao XM Identifying dysregulated pathways in cancers from pathway interaction networks BMC Bioinformatics 2012 13 126 10.1186/1471-2105-13-126 22676414 PMC3443452

[B22] Conesa A Bro R Garcia-Garcia F Prats JM Goetz S Kjeldahl K Montaner D Dopazo J Direct functional assessment of the composite phenotype through multivariate projection strategies Genomics 2008 92 373 383 10.1016/j.ygeno.2008.05.015 18652888

[B23] Antczak P Ortega F Chipman JK Falciani F Mapping drug physico-chemical features to pathway activity reveals molecular networks linked to toxicity outcome PLoS One 2010 5 e12385 10.1371/journal.pone.0012385 20811577 PMC2929951

[B24] Ponzoni I Azuaje F Augusto J Glass D Inferring adaptive regulation thresholds and association rules from gene expression data through combinatorial optimization learning IEEE/ACM Trans Comput Biol Bioinform 2007 4 624 634 17975273 10.1109/tcbb.2007.1049

[B25] Carvalho DR Freitas AA A Hybrid Decision Tree/Genetic Algorithm Method for Data Mining Inform Sciences 2004 163 13 35 10.1016/j.ins.2003.03.013

[B26] Spellman PT Sherlock G Zhang MQ Iyer VR Andres K Eisen MB Brown PO Botstein D Futcher B Comprehensive identification of cell cycle-regulated genes of the yeast Saccharomyces cerevisiae by microarray hybridization Mol Biol Cell 1998 9 3273 3297 10.1091/mbc.9.12.3273 9843569 PMC25624

[B27] Lee I Li Z Marcotte EM An improved, bias-reduced probabilistic functional gene network of baker's yeast, Saccharomyces cerevisiae PLoS ONE 2007 2 e988 10.1371/journal.pone.0000988 17912365 PMC1991590

[B28] Basco RD Segal MD Reed SI Negative Regulation of G1 and G2 by S-Phase Cyclins of Saccharomyces cerevisiae Mol Cell Biol 1995 15 5030 5042 7651421 10.1128/mcb.15.9.5030 PMC230750

[B29] Zou L Mitchell J Stillman B CDC45, a novel yeast gene that functions with the origin recognition complex and Mcm proteins in initiation of DNA Replication Mol Cell Biol 1997 17 553 563 9001208 10.1128/mcb.17.2.553 PMC231780

[B30] Uhlmann F Nasmyth K Cohesion between sister chromatids must be established during DNA replication Curr Biol 1998 8 1095 1101 10.1016/S0960-9822(98)70463-4 9778527

[B31] Michaelis C Ciosk R Nasmyth K Cohesins: chromosomal proteins that prevent premature separation of sister chromatids Cell 1997 91 35 45 10.1016/S0092-8674(01)80007-6 9335333

[B32] Paulovich AG Hartwell LH A checkpoint regulates the rate of progression through S phase in S.cerevisiae in response to DNA damage Cell 1995 82 841 847 10.1016/0092-8674(95)90481-6 7671311

[B33] Silverman SJ Petti AA Slavov N Parsons L Briehof R Thiberge SY Zenklusen D Gandhi SJ Larson DR Singer RH Metabolic cycling in single yeast cells from unsynchronized steady-state populations limited on glucose or phosphate Proc Natl Acad Sci USA 2010 107 6946 6951 10.1073/pnas.1002422107 20335538 PMC2872461

[B34] Tanaka T Nasmyth K Association of RPA with chromosomal replication origins requires an Mcm protein, and is regulated by Rad53, and cyclin- and Dbf4-dependent kinases EMBO J 1998 17 5182 5191 10.1093/emboj/17.17.5182 9724654 PMC1170846

[B35] Larsson C Nilsson A Blomberg A Gustafsson L Glycolytic Flux Is Conditionally Correlated with ATP Concentration in Saccharomyces cerevisiae: a Chemostat Study under Carbonor Nitrogen-Limiting Conditions J Bacteriol 1997 179 7243-7250 9393686 10.1128/jb.179.23.7243-7250.1997 PMC179672

[B36] Escusa S Camblong J Galan JM Pinson B Daignan-Fornier B Proteasome- and SCF-dependent degradation of yeast adenine deaminase upon transition from proliferation to quiescence requires a new F-box protein named Saf1p Mol Microbiol 2006 60 1014 1025 10.1111/j.1365-2958.2006.05153.x 16677311

[B37] Ulrich HD Regulating post-translational modifications of the eukaryotic replication clamp PCNA DNA Repair 2009 8 461 469 10.1016/j.dnarep.2009.01.006 19217833

[B38] Geoffroy MC Hay RT An additional role for SUMO in ubiquitin-mediated proteolysis Nature Rev Mol Cell Biol 2009 10 564 568 10.1038/nrm2707 19474794

[B39] Papouli E Chen S Davies AA Huttner D Krejci L Sung P Ulrich HD Crosstalk between SUMO and ubiquitin on PCNA is mediated by recruitment of the helicase Srs2p Mol Cell 2005 19 123 133 10.1016/j.molcel.2005.06.001 15989970

[B40] Pfander B Moldovan GL Sacher M Hoege C Jentsch S SUMO-modified PCNA recruits Srs2 to prevent recombination during S phase Nature 2005 436 428 433 15931174 10.1038/nature03665

[B41] Stelter P Ulrich HD Control of spontaneous and damage-induced mutagenesis by SUMO and ubiquitin conjugation Nature 2003 425 188 191 10.1038/nature01965 12968183

[B42] Haracska L Torres-Ramos CA Johnson RE Prakash S Prakash L Opposing effects of ubiquitin conjugation and SUMO modification of PCNA on replicational bypass of DNA lesions in Saccharomyces cerevisiae Mol Cell Biol 2004 24 4267 4274 10.1128/MCB.24.10.4267-4274.2004 15121847 PMC400445

[B43] Blalock EM Geddes JW Chen KC Porter NM Markesbery WR Landfield PW Incipient Alzheimer's disease: microarray correlation analyses reveal major transcriptional and tumor suppressor responses Proc Natl Acad Sci USA 2004 101 2173 2178 10.1073/pnas.0308512100 14769913 PMC357071

[B44] Liang WS Dunckley T Beach TG Grover A Mastroeni D Walker DG Caselli RJ Kukull WA McKeel D Morris JC Gene expression profiles in anatomically and functionally distinct regions of the normal aged human brain Physiol Genomics 2007 28 311 322 17077275 10.1152/physiolgenomics.00208.2006 PMC2259385

[B45] Bubber P Haroutunian V Fisch G Blass JP Gibson GE Mitochondrial abnormalities in Alzheimer brain: Mechanistic implications Annals of Neurology 2005 57 695 703 10.1002/ana.20474 15852400

[B46] Rex Sheu KF Kim YT Blass JP Weksler ME An immunochemical study of the pyruvate dehydrogenase deficit in Alzheimer's disease brain Annals of Neurology 1985 17 444 449 10.1002/ana.410170505 4004169

[B47] Kou J Kovacs GG Höftberger R Kulik W Brodde A Forss-Petter S Hönigschnabl S Gleiss A Brügger B Wanders R Just W Budka H Jungwirth S Fischer P Berger J Peroxisomal alterations in Alzheimer's disease Acta Neuropathol 2011 122 271 83 10.1007/s00401-011-0836-9 21594711 PMC3168371

[B48] Munoz L Ammit AJ Targeting p38 MAPK pathway for the treatment of Alzheimer's disease Neuropharmacology 2010 58 561 568 10.1016/j.neuropharm.2009.11.010 19951717

[B49] Kim EK Choi EJ Pathological roles of MAPK signaling pathways in human diseases Biochim Biophys Acta 2010 1802 396 405 10.1016/j.bbadis.2009.12.009 20079433

[B50] Kou J Kovacs GG Höftberger R Kulik W Brodde A Forss-Petter S Hönigschnabl S Gleiss A Brügger B Wanders R Just W Budka H Jungwirth S Fischer P Berger J Peroxisomal alterations in Alzheimer's disease Acta Neuropathol 2011 122 271 83 10.1007/s00401-011-0836-9 21594711 PMC3168371

[B51] Lizard G Rouaud O Demarquoy J Cherkaoui-Malki M Iuliano L Potential roles of peroxisomes in Alzheimer's disease and in dementia of the Alzheimer's type J Alzheimers Dis 2012 29 241 54 22433776 10.3233/JAD-2011-111163

[B52] Grammas P Sanchez A Tripathy D Luo E Martinez J Vascular signaling abnormalities in Alzheimer disease Cleve Clin J Med 2011 78 Suppl 1 S50 21972332 10.3949/ccjm.78.s1.09

[B53] Rosenstein JM Krum JM Ruhrberg C VEGF in the nervous system Organogenesis 2010 6 107 114 10.4161/org.6.2.11687 20885857 PMC2901814

[B54] Caltagarone J Jing Z Bowser R Focal Adhesions Regulate Aβ Signaling & Cell Death in Alzheimer's Disease Biochim Biophys Acta 2007 1772 438 445 10.1016/j.bbadis.2006.11.007 17215111 PMC1876750

[B55] Kehoe PG The renin-angiotensin-aldosterone system and Alzheimer's disease? J Renin Angiotensin Aldosterone Syst 2003 4 80 93 10.3317/jraas.2003.017 12806589

[B56] Amouyel P Richard F Berr C David-Fromentin I Helbecque N The renin angiotensin system and Alzheimer's disease Ann N Y Acad Sci 2000 903 437 441 10.1111/j.1749-6632.2000.tb06395.x 10818534

[B57] Weisgraber KH Mahley RW Human apolipoprotein E: the Alzheimer's disease connection FASEB J 1996 10 1485 1494 8940294 10.1096/fasebj.10.13.8940294

[B58] Mahley RW Huang Y Apolipoprotein (apo) E4 and Alzheimer's disease: unique conformational and biophysical properties of apoE4 can modulate neuropathology Acta Neurologica Scandinavica 2006 114 s185 8 14 10.1111/j.1600-0404.2006.00679.x 16866905

[B59] Henderson ST High carbohydrate diets and Alzheimer's disease Medical Hypotheses 2004 62 689 700 10.1016/j.mehy.2003.11.028 15082091

[B60] Rébora K Desmoucelles C Borne F Pinson B Daignan-Fornier B Yeast AMP pathway genes respond to adenine through regulated synthesis of a metabolic intermediate Mol Cell Biol 2001 21 7901 12 10.1128/MCB.21.23.7901-7912.2001 11689683 PMC99957

[B61] Lai Y Wu B Chen L Zhao H A statistical method for identifying differential gene-gene co-expression patterns Bioinformatics 2004 20 3146 55 10.1093/bioinformatics/bth379 15231528

[B62] Hu R Qiu X Glazko G Klebanov L Yakovlev A Detecting intergene correlation changes in microarray analysis: a new approach to gene selection BMC Bioinformatics 2009 10 20 10.1186/1471-2105-10-20 19146700 PMC2657217

[B63] Watson-Haigh NS Kadarmideen HN Reverter A PCIT: an R package for weighted gene co-expression networks based on partial correlation and information theory approaches Bioinformatics 2010 26 411 3 10.1093/bioinformatics/btp674 20007253

[B64] Efron B Tibshirani R On testing the significance of sets of genes Ann Appl Stat 2007 1 107 129 10.1214/07-AOAS101

[B65] Langfelder P Horvath S WGCNA: an R package for weighted correlation network analysis BMC Bioinformatics 2008 9 559 10.1186/1471-2105-9-559 19114008 PMC2631488

[B66] Mostafavi S Ray D Warde-Farley D Grouios C Morris Q GeneMANIA: a real-time multiple association network integration algorithm for predicting gene function Genome Biol 2008 9 Suppl 1 S4 10.1186/gb-2008-9-s1-s4 18613948 PMC2447538

[B67] Huang D Friesen H Andrews B PHO85, a multifunctional cyclin-dependent protein kinase in budding yeast Molecular Microbiology 2007 66 303 314 10.1111/j.1365-2958.2007.05914.x 17850263

[B68] Hudson NJ Reverter A Dalrymple BP A differential wiring analysis of expression data correctly identifies the gene containing the causal mutation PLoS Comput Biol 2009 5 e1000382 10.1371/journal.pcbi.1000382 19412532 PMC2671163

[B69] Hudson NJ Dalrymple BP Reverter A Beyond differential expression: the quest for causal mutations and effector molecules BMC Genomics 2012 13 356 10.1186/1471-2164-13-356 22849396 PMC3444927

[B70] Tarazona S Prado-López S Dopazo J Ferrer A Conesa A Variable selection for multifactorial genomic data Chemometr Intell Lab 2012 110 113 122 10.1016/j.chemolab.2011.10.012

[B71] Mitchell TM Machine Learning 1997 Boston: WCB/McGraw-Hill

[B72] Kohani R Wrappers for Performance Enhancement and Oblivious Decision Graphs PhD dissertation 1995 Computer Science Dept., Stanford Univ., USA

[B73] García-Alcalde F García-López F Dopazo J Conesa A Paintomics: a web based tool for the joint visualization of transcriptomics and metabolomics data Bioinformatics 2011 27 137 139 10.1093/bioinformatics/btq594 21098431 PMC3008637

[B74] Fellenberg K Hauser NC Brors B Neutzner A Hoheisel JD Vingron M Correspondence analysis applied to microarray data Proc Natl Acad Sci USA 2001 98 10781 10786 10.1073/pnas.181597298 11535808 PMC58552

