# Supplementary material for: Pathway network inference from gene expression data
Source: BMC Syst Biol. 2014 Mar 13;8(Suppl 2):S7. doi: 10.1186/1752-0509-8-S2-S7 (PMC4101702; doi:10.1186/1752-0509-8-S2-S7)
Supplement: Additional file 1 — Figure S1. Performance of control parameters for opposite rules. Average correlation among Pathway Profiles of opposite rules (left axis, red polygon) and percentage of opposite links with same simulated expression profile SEP (right axis, blue polygon) in the resulting network, as a function of the accuracy threshold. The upper and lower border of polygons indicate the range of variation at different alpha values. Noise level was set at 0.01. Figure S2. Association score enrichment against random pathways. For each pathway association rule R present in the YCCPN, a total of 100 random pathway associations with the same cardinality pattern as R were generated, the ASp values computed and the percentile position of the ASp of R in its reference distribution was obtained. The cardinality pattern of a rule is defined by three values: the amount of genes contained in each pathway linked by the rule, and the number of shared genes between both pathways. This analysis revealed that most (63% of the links) of the rules obtained by the PANA method are located in the 20% percentile of the 100 random trials of their gene cardinality pattern. In particular, the average bASn of network integrated by the random links is low (28.50) in comparison with the bASn of the YCCPN (123.61). The difference between YCCPN bASn and the random bASn was statistically significant (t-test p-value < 0.05). Figure S3. Independence of the ASp score of the pathway size. Relationship between pathway association scores (bASp and mASp values) and the number of genes in the left (dot) and right (cross) pathways. Lack of correlation is observed in all cases. Table S1. Simulated expression profiles (SEP). Temporal expression patterns defined for the generation of simulated time series for the artificial pathways. Table S2. Network size (number of pathway associations) inferred using different accuracy and alpha values in the Yeast Cell Cycle network obtained by PANA. [file 1752-0509-8-S2-S7-S1.doc]

**ADDITIONAL FILE 1**

**Figure S1. Performance of control parameters for opposite rules**. Average correlation among Pathway Profiles of opposite rules (left axis, red polygon) and percentage of opposite links with same simulated expression profile SEP (right axis, blue polygon) in the resulting network, as a function of the accuracy threshold. The upper and lower border of polygons indicate the range of variation at different alpha values. Noise level was set at 0.01.

**
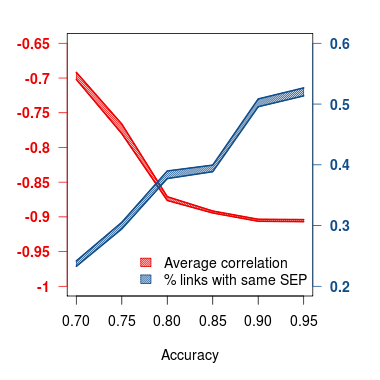
**

**Figure S2. Association score enrichment against random pathways.** For each pathway association rule R present in the YCCPN, a total of 100 random pathway associations with the same cardinality pattern as R were generated, the ASp values computed and the percentile position of the ASp of R in its reference distribution was obtained. The cardinality pattern of a rule is defined by three values: the amount of genes contained in each pathway linked by the rule, and the number of shared genes between both pathways. This analysis revealed that most (63% of the links) of the rules obtained by the PANA method are located in the 20% percentile of the 100 random trials of their gene cardinality pattern. In particular, the average *b*ASn of network integrated by the random links is low (28.50) in comparison with the *b*ASn of the YCCPN (123.61). The difference between YCCPN *b*ASn and the random *b*ASn was statistically significant (t-test p-value < 0.05).

**
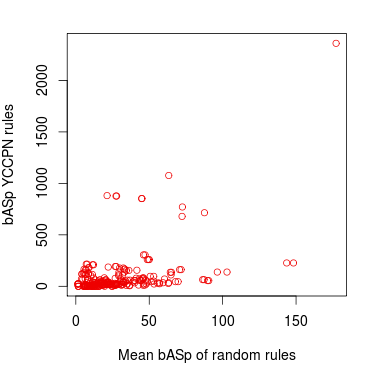
**

**Figure S3. Independence of the ASp score of the pathway size.** Relationship between pathway association scores (*b*ASp and *m*ASp values) and the number of genes in the left (dot) and right (cross) pathways. Lack of correlation is observed in all cases.


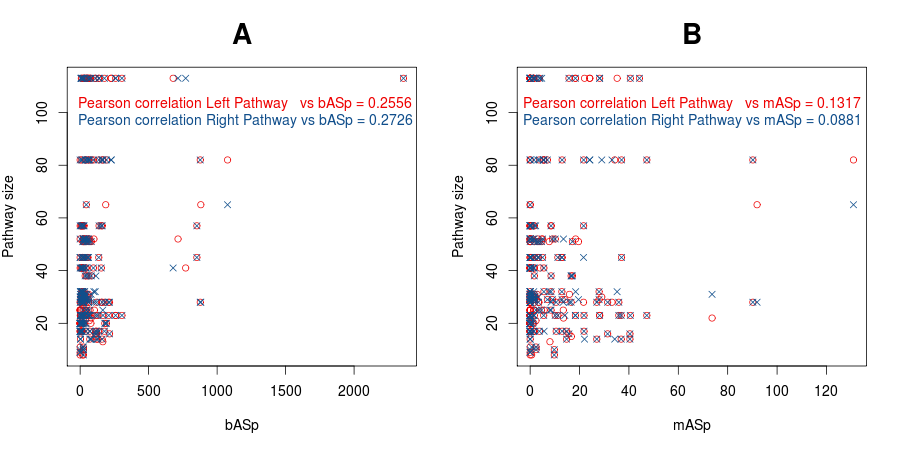


**Table S1. Simulated expression profiles (SEP). Temporal expression patterns defined for the generation of simulated time series for the artificial pathways.**

| **Number** | **Description** |
| --- | --- |
| 1 | Continuous induction |
| 2 | Continuous repression |
| 3 | Transitory induction at early time points |
| 4 | Transitory induction at late time points |
| 5 | Transitory induction at early and late time points |
| 6 | Induction at early time point and maintained constant |
| 7 | Induction at late time point and maintained constant |

**Table S2.** Network size (number of pathway associations) inferred using different accuracy and alpha values in the Yeast Cell Cycle network obtained by PANA.

| **Accuracy** | **α=0.05** | **α=0.01** | **α=0.005** | **α=0.001** |
| --- | --- | --- | --- | --- |
| 0.70 | 7094 | 3833 | 3288 | 1670 |
| 0.75 | 4201 | 2323 | 1983 | 1013 |
| 0.80 | 2645 | 1463 | 1241 | 654 |
| 0.85 | 1665 | 918 | 767 | 438 |
| 0.90 | 847 | 514 | 415 | 252 |
| 0.95 | 219 | 152 | 130 | 83 |
